# Rapid Education Event: A Streamlined Approach to Ultrasound Guided Nerve Block Procedural Training

**DOI:** 10.7759/cureus.34080

**Published:** 2023-01-23

**Authors:** Claire L Paulson, Kristine L Schultz, Daniel Kim, Kevin R Roth, Hanna R Warren

**Affiliations:** 1 Department of Emergency and Hospital Medicine/USF Morsani College of Medicine, Lehigh Valley Health Network, Bethlehem, USA

**Keywords:** continuing medical education, curriculum, ultrasound, rapid education event, nerve block

## Abstract

In the Emergency Medicine Residency setting, procedural ultrasound education often takes place at the bedside when the procedure becomes clinically necessary. As ultrasound technology and its applications continue to gain more importance, there is a greater need for effective and standardized educational models for teaching ultrasound-guided procedures. This pilot program aimed to demonstrate that residents and attending physicians can achieve procedural competence in fascia iliaca nerve block following a rapid and compact procedural education event. Our curriculum covered anatomy identification, procedural knowledge, and technical skills of probe manipulation. After completing our new curriculum, more than 90% of participants demonstrated adequate learning through the pre- and post-assessments and direct observation of procedural performance on a gel phantom model.

## Introduction

The advancement of diagnostic and procedural Point-of-Care Ultrasound (POCUS) demands that attending physicians and residents adopt ultrasound-guided procedures into their patient care. POCUS guidance in various procedures including regional anesthesia/nerve blocks has been shown to improve procedural safety and efficacy as well as patient satisfaction [1-3]. Many practicing attending physicians have not been formally trained in these relatively new procedures during their residency and it is essential that we create an effective and standardized training model for ultrasound-guided procedures. We developed a model called a Rapid Educational Event (REE), which was designed to expand the procedural skill set of ultrasound-guided nerve blocks for attending physicians and residents in the Department of Hospital and Emergency Medicine.

This educational intervention aimed to assess the effectiveness of the REE in attaining physician and resident competency in ultrasound-guided nerve block, specifically the fascia iliaca block. The goal was to have at least 90% of the learners demonstrate knowledge of the fascia iliaca block by passing the post-assessment with a score of 80% or higher and demonstrate procedural competency by completing a directly observed mock fascia iliaca block on a gel phantom.

This article was previously presented, in part, as an abstract at the 2022 American College of Emergency Physicians Teaching Fellowship Meeting on August 4-9, 2022.

## Technical report

The REE educational model coupled lecture-based instruction with hands-on learning of the procedure to maximize training in the allotted time. It was submitted and approved for CME credits for those eligible for CME (not trainees). It was reviewed by the network IRB and determined to not be human subject research and IRB oversight was therefore waived for the project. Three REE sessions were executed over a two-month period in the spring of 2022 to reach the various learners in the department. Attending core faculty were approached and invited to participate, and inclusion was voluntary. The session for trainees was held at grand rounds and the residents were required to attend unless they met other criteria that prohibited attendance (for example illness, or duty hour violation). Learners were provided in advance with a video detailing the procedure and they were instructed to watch it prior to the REE. They also completed a pre-course assessment to establish their knowledge pertaining to fascia iliaca block. The training was designed to cumulatively take approximately one hour per participant. The pre-class video was ten minutes in length, the PowerPoint facilitated lecture at the hands-on session was about 15 minutes, and practice at the stations was approximately 30 minutes.

**Figure 1:**
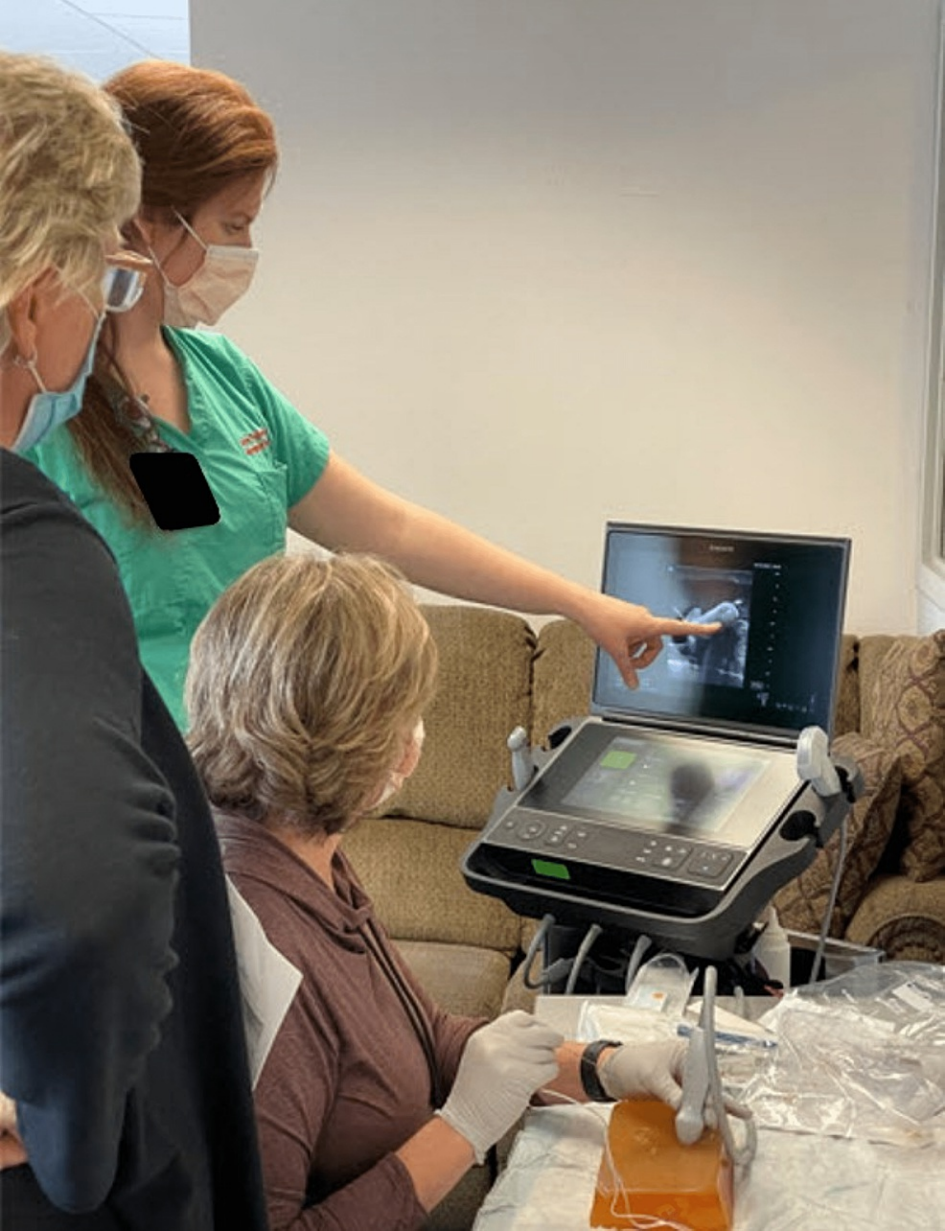
Learner practicing placement of echogenic needle under ultrasound guidance on gel phantom models. Photo Credit of Timothy J. Batchelor, MD.

The REE also included a hands-on session with live standardized patients. Learners were given a pre-assessment to assess their understanding of previously supplied material regarding the procedure. During the first portion of the class, short lectures were given to reaffirm learners’ knowledge and to answer any questions. The following time was spent completing the hands-on portion of the learning. Learners were divided into groups and proceeded through two stations. At the first station, learners practiced using ultrasound to identify the appropriate anatomy of the femoral artery, femoral vein, and femoral nerve in standardized patients. They were given ample time to scan each patient and were given tips and tricks by the instructors at each station. At the second station, learners practiced placing echogenic needles under ultrasound guidance on gel phantom models (Figure 1). The gel phantoms were designed to simulate the femoral nerve, femoral artery, and femoral vein. Thus, the learners were able to actively scan the gel phantoms and practice inserting the needle tip to the correct location.

Once each learner completed both stations, they proceeded to the post-assessment. They were provided with a written post- assessment that was graded by the course faculty. They also completed a directly observed mock nerve block on a model to demonstrate their understanding of the procedure. For this portion of the assessment, we graded based on a pre-designed checklist of actions (Table 1).

**Table 1:** The action checklist that all learners were graded on when practicing a mock nerve block.

ITEM	PERFORMED (circle one)	SEQUENCE ERROR (check √)
Joint Commission Timeout		
Verbalized that time out performed/ consent obtained	YES NO	
Equipment and Set-up/ Medication Preparation		
Verbalize that calculates maximum dosage of anesthetic, perform pre-procedure checklist (Confirm IV access, Pt connected to telemetry, US prepared, Block kit complete, Lipid emulsion kit available)	YES NO	
Ultrasound Set Up		
Notes correct orientation of probe	YES NO	
Applies probe cover/ tegaderm	YES NO	
Positioning		
Places in supine/ frog leg position (If not already done so)	YES NO	
Anatomy		
Identify landmarks and the insertion point (Verbalize and show)	YES NO	
Identifies Nerve, Artery, and Vein on US screen	YES NO	
Sterile Technique		
Hand washing (With soap and water or chemical)	YES NO	
Cleanses skin using Chloraprep scrub in back-and-forth motion for 30 seconds. Allow skin to dry	YES NO	
Drape to create sterile field with half sheet/ towel	YES NO	
Uses sterile gel	YES NO	
Procedure		
Correctly identifies proper needle insertion site using US	YES NO	
Anesthetize skin and soft tissue (expected route of insertion) with 1% Lidocaine and 25 gauge needle	YES NO	
Aspirates while advancing	YES NO	
Using NB needle, injects saline to confirm proper needle placement	YES NO	
Successfully injects near nerve	YES NO	

This checklist was designed to objectively assess procedural motor skill comprehension. It was adapted from one utilized in our central line course for new residents and subsequently underwent internal peer review by our Emergency Ultrasound Faculty. The checklist was additionally streamlined after our first trial session of the event based on both learner and educator feedback. The final checklist was again approved by all course faculty prior to implementation.

The responses of the learners from pre- and post- assessments were automatically de-identified. These responses were transmitted to the Department of Emergency and Hospital Medicine (DOEHM) scholarly activities study coordinator, who assigned each survey a study ID number and entered the data into a secure spreadsheet. Descriptive statistics were used to describe the entire sample. Pass/fail rates were described using counts and percentages. The mean and standard deviation were used to describe assessment and checklist scores. Differences between pre- and post-test scores were assessed using a t-test. A Chi-square test was used to assess differences in pass/fail rates between pre- and post-testing. All analyses were two-tailed, with alpha set at 0.05.

Results

Subjectively, our students reported feeling confident and competent in performing this procedure after completing this educational course. Their competence was objectively measured as all fifty-nine participating learners passed their observed mock nerve block evaluation and scored 100% on the checklist of observed nerve block on gel phantom. Aggregate pre-assessment data from all three sessions in which attending physicians and residents participated, showed that the pre-assessment pass rate was 74.5% (44/59) and that of post-assessment was 91.5% (54/59). All 59 (100%) of the learners passed the mock fascia iliaca block on a gel phantom model. The analysis of data collected from this course supports the effectiveness of the REE (Table 2).

**Table 2:** Data gathered from learners, aggregated across all education sessions. A minimum score of 80% was required to pass each assessment. N = 59 for all data presented in this table. SD= Standard Deviation ^a^t-test used to calculate p-value ^b^Chi-Square test used to calculate p-value, Degrees of freedom = 1

	Pre-Education Assessment	Post-Education Assessment	P-Value
Mean Test Score Mean % ± SD	84.18% ± 20.76	93.17% ± 8.64	<0.0001^a^
Number of learners with passing score	44	54	0.0141^b^
Learner checklist score	N/A	100%	N/A

## Discussion

The use of ultrasound (US)-guided fascia iliaca nerve block in the ED setting has become increasingly common and is well-documented [4-6]. While there is a scarcity of data concerning the development of a training program for this procedure, the American College of Emergency Physicians (ACEP) has published guidelines that provide a general outline for performing educational sessions with emergency physicians [7]. The REE model adheres to those guidelines by providing a lecture portion, followed by hands-on training, all while maintaining a low student-to-teacher ratio. Our goal in implementing this educational program was to decrease the amount of time that a learner would need to spend on training, in order to become proficient in a new procedure.

To that end, our department developed and implemented a training program using methods that allowed the learners more flexibility and decreased their overall time commitment without reducing the effectiveness of the training. For the didactic segment of the program, we used a hybrid educational model in which the learner was required to watch a 10-minute video detailing the procedure prior to the REE. On the day of the event, a 15-minute in-person lecture was given to reinforce the lessons learned in the video. Following the lecture, learners practiced visualizing the relevant structures using live standardized patients. Finally, they demonstrated their procedural skills using gel phantom models to simulate a live patient, spending just 30 minutes at each station. Previously published curricula typically involve several training sessions held over multiple days to teach similar US-guided procedures and skills [8-10].

Comparison of pre- and post-assessment test scores and the 100% pass rate during the hands-on portion of the training both support the conclusion that the REE model is an effective training program. We believe that the REE is a model that could be adapted for use by other institutions with relative ease. By decreasing the learners’ time commitment, this program is more accessible to the time-pressed physician and is perceived as less onerous than a multi-day CME course.

We look forward to continuing to measure the impact of this course. Future directions of study include monitoring the number of fascia iliaca blocks completed successfully by our attending physicians and residents in the clinical domain. We also plan to implement this REE design for further training in the Department of Emergency and Hospital Medicine. Future research directions would include reproducing the same study at other EM residency programs (multisite engagement) and additionally, evaluating outcomes for the same REE applied to other USG-guided blocks and bedside procedures.

## Conclusions

Overall, the REE was very well received by attending physicians and residents. The pre-course work, including the pre-recorded lectures and the pre-assessment, was completed on the learner’s own time. This allowed more time for the learners during the hands-on event to practice procedural skills. Learning ultrasound-guided procedures requires repetition, and our REE allowed our learners to practice on multiple live standardized patients and simulation gel phantoms, both of which effectively simulated real-life clinical scenarios.
